# Functional outcomes after intramedullary nailing (C-Nail®) of severe calcaneal fractures with mean follow-up of 36 months

**DOI:** 10.1007/s00068-023-02433-3

**Published:** 2024-01-16

**Authors:** Philipp Schippers, Rasmus Engels, Dominik Benning, Sebastian Fischer, Felix Wunderlich, Yama Afghanyar, Charlotte Arand, Michael Nienhaus, Philipp Drees, Erol Gercek, Raphael Küchle

**Affiliations:** 1 Department of Orthopedics and Traumatology, University Medical Center of the Johannes Gutenberg University Mainz, 55131 Mainz, Germany; 2 Medical Point Chirurgie Wiesbaden, 65183 Wiesbaden, Germany; 3 Department of Orthopaedics and Trauma Surgery, Reconstructive Surgery, Hand Surgery, Plastic Surgery and Burn Medicine, German Armed Forces Central Hospital, Koblenz, Germany; 4 Department of Foot and Ankle Surgery, Berufsgenossenschaftliche Unfallklinik Frankfurt Am Main, 60389 Frankfurt, Germany

**Keywords:** Foot trauma, Calcaneal fractures, C-Nail, Intramedullary nailing, PROMs, Return to sports, Return to work

## Abstract

**Purpose:**

Calcaneal fractures (CFs) are rare but potentially debilitating injuries. Apart from the open, far lateral or sinus tarsi approach, operative treatment can be performed minimally invasive and percutaneously with intramedullary nailing. In this study, we sought to investigate the functional outcome of severe CFs treated with the C-Nail® implant.

**Methods:**

Twenty-two CFs (9 × Sanders III and 8 × Sanders IV), operated between 2016 and 2019, were followed up with a mean duration of 36 (± 11) months. The AOFAS score, pre- and postoperative Böhler angles, wound healing disorders, and patient-reported outcome measures (PROMs) like pain levels and return to work/sport levels were assessed.

**Results:**

The mean AOFAS score was 72.0 (± 9.8). Four patients sustained wound healing disorders, yet no implant-associated surgical revision was required. Fifty percent of patients were pain-free within 1 year. Within 1 year, about 50% of the patients could return to sports, and about 80% of the patients could return to work. PROMs and functional results align with those from other implants reported in the literature.

**Conclusion:**

Intramedullary nailing of severe CFs with the C-Nail® implant can be considered a safe treatment alternative that yields acceptable results at mid-terms.

## Introduction

Calcaneal fractures (CFs) are rare injuries [1], mainly occurring in middle-aged men [2]. Historically, CFs were called “Lover’s fracture” or “Casanova fracture,” referring to a suitor who sustained this type of fracture after jumping from his inamorata’s balcony to avoid detection [3]. Nowadays, the most common etiology remains the direct axial force due to a fall from height [1]. Calcaneal fractures can have devastating consequences for the patient and cause long-term disability with a high socio-economic burden [4].

While there is an ongoing debate about whether CFs should be treated operatively or conservatively, some data suggest that operative treatment yields better results [5–9]. Open reduction and internal fixation with direct visualization of the fracture appears to be the “gold standard” [10]. The treatment aims to restore calcaneal width, height, shape, and alignment. The Böhler angle, defined by two lines tangent to the calcaneus anterior and superior aspect, ranges between 20 and 40° in healthy subjects [11]. The Böhler angle correlates with the severity of a CF, and its restoration is vital for the outcome [12].

Wound healing disorders are amongst the most common and feared complications after open surgery, with a prevalence of 1.3% for deep and up to 27% for superficial infections [13, 14]. Important risk factors are a high BMI, smoking, diabetes, and the severity of the fracture [15, 16]. Consequently, several techniques were developed to minimize the operative trauma and reduce the size of the wound to lower postoperative complications. Amongst these are minimally invasive approaches like the sinus tarsi approach, arthroscopically-assisted reduction and fixation, or percutaneous techniques like intramedullary nailing [17–20].

The first calcaneal intramedullary nail was implanted in 1882 by Dr. Gussenbauer, but the concept was abandoned until Goldzak et al. introduced an intramedullary nail for CFs in 2012 [21]. The goal was to find a treatment alternative with less soft tissue damage. In a prospective study, Simon et al. published results from 69 patients treated with the Calcanail® (FH Orthopedics, Heimsbrunn, France), yielding promising results [22]. Another implant, the C-Nail® (Medin, Nov. Město n. Moravě, Czech Republic), was introduced by Zwipp et al. in 2016 and showed good results in a multicenter study involving 106 patients yielding a mean AOFAS score of 89.5 6 months postoperatively [23]. Both Calcanail® and C-Nail® proved to be at least equally strong as the locking plate in a cadaveric biomechanical study [24]. Although Calcanail® and C-Nail® share similarities in their design, the implants achieve reduction and fixation through different mechanisms. While the Calcanail® is stabilized by two screws and relies on indirect reduction of the posterior facet, the C-Nail® requires direct visualization of the joint space through a sinus tarsi approach and several multi-directional screws [25]. There is still no clarity on whether the locking plate or intramedullary nailing is superior [26].

In 2019, Fourgeaux et al. and Herlyn et al. published functional outcomes of CFs treated with the Calcanail® implant. Fourgeaux et al. investigated 26 patients with a mean follow-up of 33 months. The fracture severity was balanced, with 46% having a Sanders II and 53% having a Sanders III/IV fracture. The mean AOFAS score was 79, and return to work was achieved after a mean duration of 6.5 months [27]. Herlyn et al. examined 20 patients prospectively with a mean follow-up of 11.3 months, achieving a mean AOFAS score of 71.6. The mean duration for return to work was 15.8 weeks; nine patients could not return to their previous sports activities. Results were matched with historical data of 20 feet treated with a locking plate through an extended lateral approach [28]. Finally, Steinhausen et al. performed a retrospective matched pair analysis of one hundred and one CFs treated with a locking plate or the C-Nail®. Sixty-two percent of patients receiving the C-Nail® had a Sanders IV fracture. The mean AOFAS score was 79.4 after 6 months without differences between the groups. However, there were significantly fewer complications, and patients were earlier able to perform full-weight bearing after receiving the C-Nail®. Finally, the authors suggest a more prolonged follow-up [29].

Most studies investigated the Calcanail® implant [22, 27, 28, 30–32], while fewer studies investigated functional outcomes of the C-Nail® implant with a follow-up of only 1 year or even less [23, 26, 29].

To our best knowledge, no data on return to work and return to sports levels is available for the C-Nail® implant, especially in severe (Sanders ≥ III) fractures with a longer follow-up. This study aimed to investigate the functional outcome at mid-terms after treatment of severe CFs using the C-Nail® implant in a cohort with mainly severe fractures.

## Methods

### Surgical technique

**Fig. 1:**
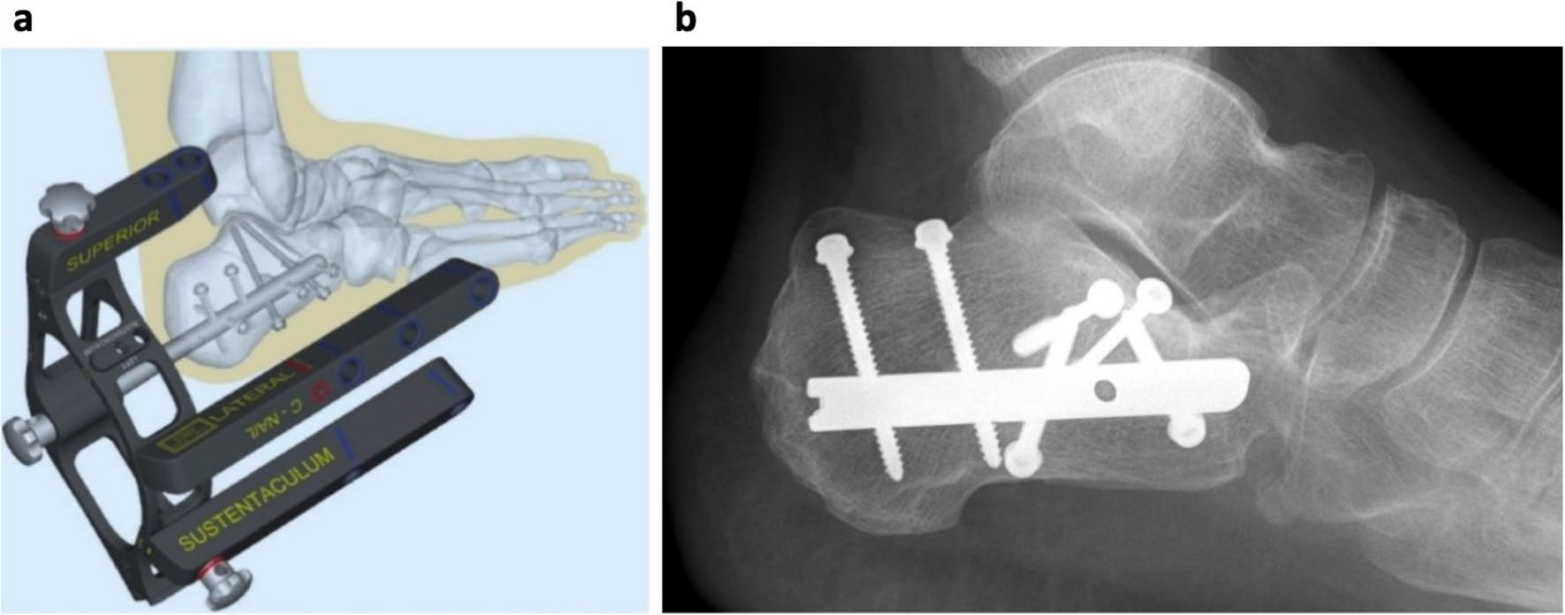
*C-Nail® target instrument and exemplary postoperative X-ray*. **a** The sketch of the C-Nail® implant with the target instrument. Image with kind permission from Medin (Nov. Město n. Moravě, Czech Republic). **b** An exemplary postoperative X-ray. Here, two screws were placed through a sinus tarsi approach and four locking screws were placed through the nail. A drain was inserted at the sinus tarsi incision

Patients were positioned laterally on the healthy side, and the fractured foot was placed on a soft pad. The posterior joint facet was reconstructed through a sinus tarsi approach, and osteosynthesis was performed with two cancellous screws. After restoration of the joint facet, the C-Nail® implant was introduced percutaneously below the insertion of the Achilles tendon in a posterior-anterior direction after probing with a K-wire. With the help of the three arms of the target instrument, the interlocking screws were placed through the nail, starting with the screw for the sustentaculum tali (Fig. 1a). Passive motion was started on day two postoperatively. On day five, partial weight-bearing was started. After 6 to 8 weeks, full weight-bearing was initiated.

### Study design

**Fig. 2:**
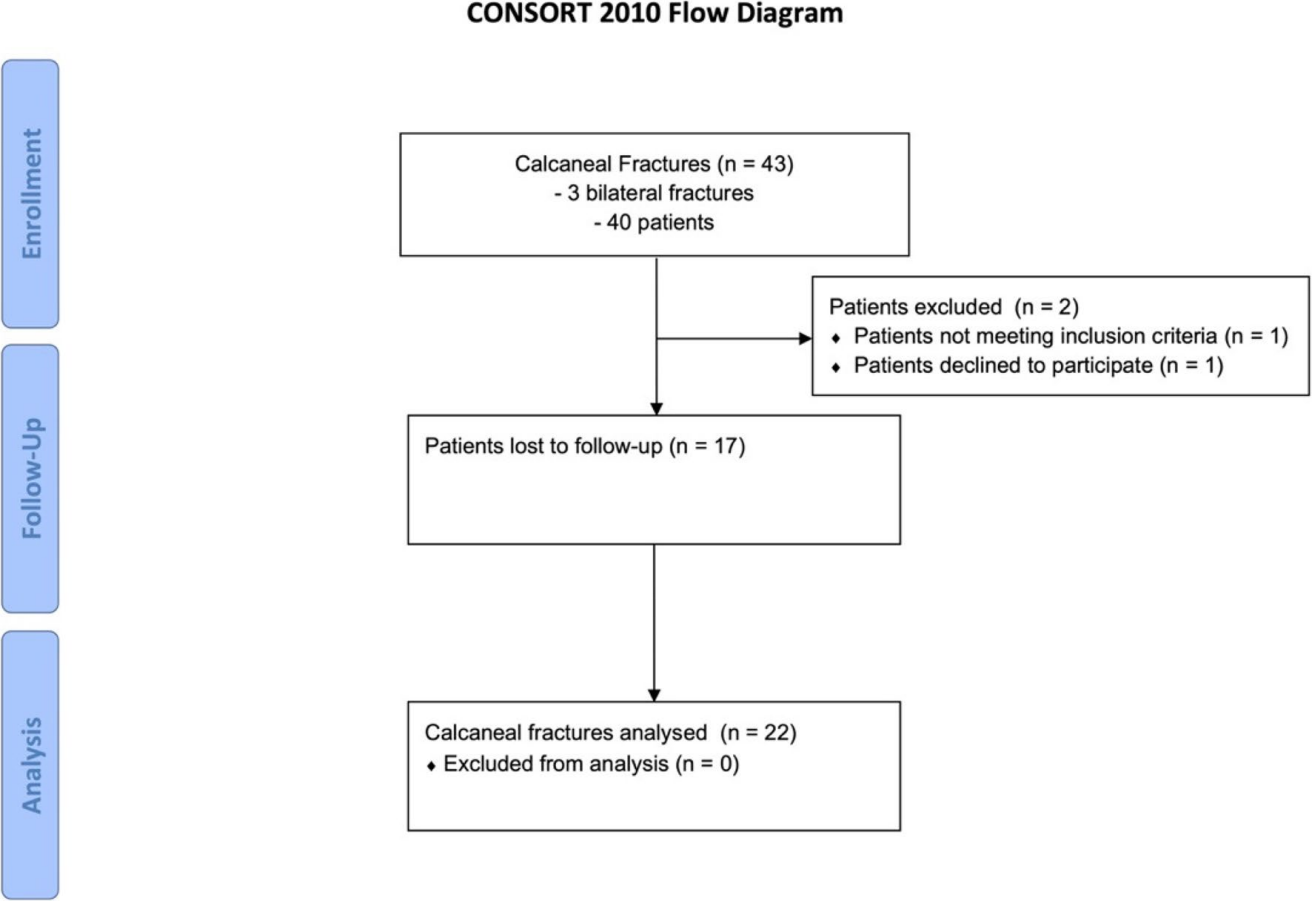
Study flow chart

The C-Nail® implant was introduced in our department in 2016. Between 2016 and 2019, 43 CFs were treated using the C-Nail® implant. Three patients presented with bilateral CFs. After receiving IRB approval, all 40 patients were contacted and invited to a follow-up exam. One patient suffered from dementia and was thus not able to consent to participate; one patient refused participation. Seventeen patients were lost to follow-up. Finally, 22 CFs were examined (Fig. 2). The final population contained 20% women and 80% men.

### Follow-up examination

Mean follow-up took part 36 months (± 11) post-surgery. Patients were asked to fill out the AOFAS score [33] and a self-constructed questionnaire for patient-reported outcome measures (PROMs), as shown in Table 1. Patients who had bilateral fractures were asked to undergo AOFAS scoring and PROMs individually for both feet.

**Table 1:** Patient questionnaire

Question	Possible answers
Wound healing disorders?	- No - Yes
Local irritations? (reddening, itching, but intact wound/scar)	- No - Yes
Change of shoes?	- No - Yes
Time needed to be pain-free?	- Less than 3 months - Less than 6 months - Less than 12 months - More than 12 months - Never
Time needed to return to full-weight bearing?	- Less than 6 weeks - Less than 3 months - Less than 6 months - Less than 12 months - More than 12 months - Never
Time needed to return to sports?	- Less than 3 months - Less than 6 months - Less than 12 months - More than 12 months - Never
Time needed to return to work?	- Less than 3 months - Less than 6 months - Less than 12 months - More than 12 months - Never

At follow-up, patients filled out the above-shown questionnaire

### Radiographic analysis

Preoperative Böhler angles were measured on preoperative lateral view X-rays. Postoperatively, patients received another lateral view X-ray of the fractured calcaneus to determine the postoperative Böhler angle (Fig. 1b). The change in the Böhler angle was used to assess the quality of the reduction.

### Statistical analysis

Statistical analysis was performed using Prism 10 (GraphPad Software Inc., Boston, USA). Spearman’s correlations were calculated between the AOFAS score and the PROMs; correlation coefficients higher than *r* = 0.4 were interpreted as “moderate,” and coefficients higher than 0.7 were interpreted as “strong” [34].

## Results

**Fig. 3:**
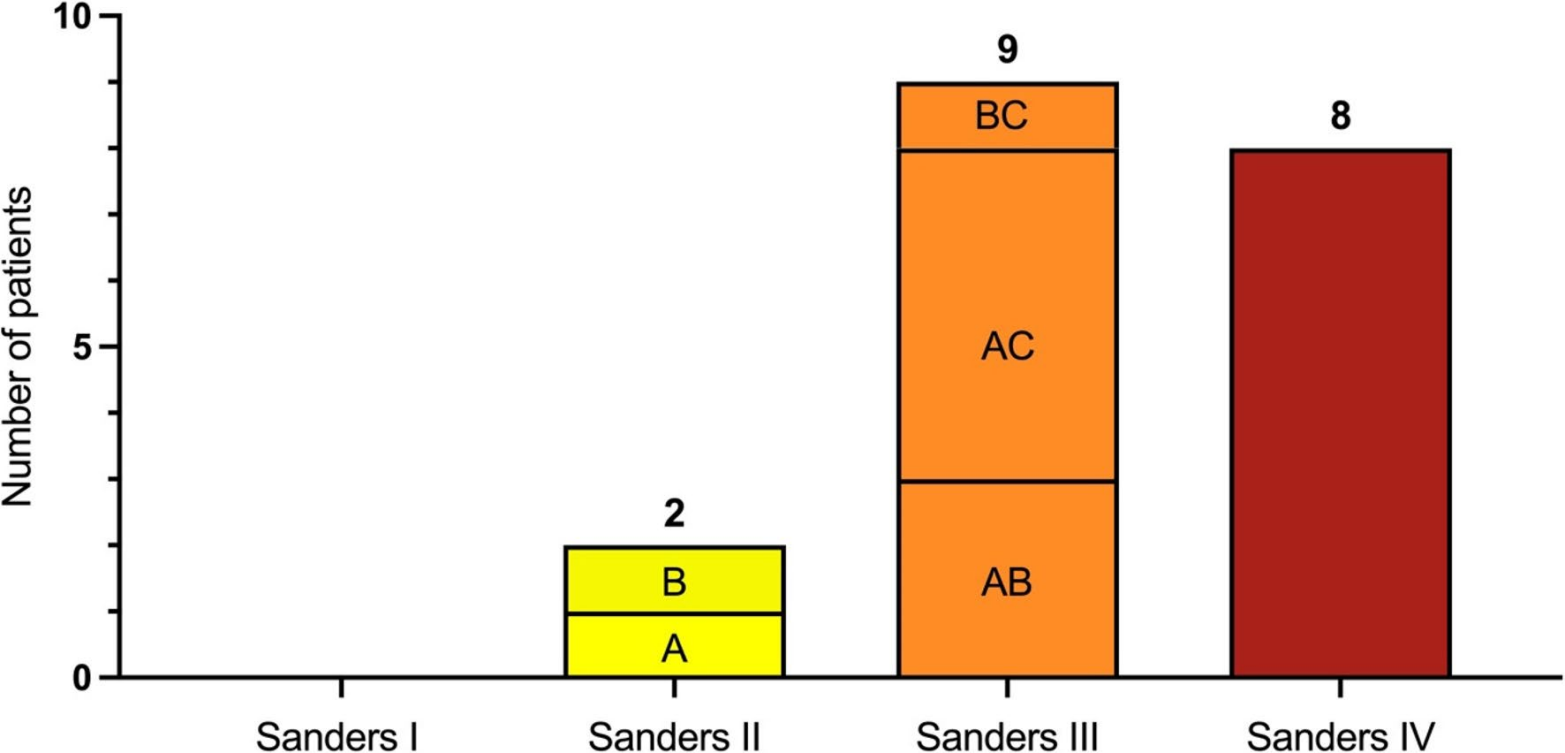
Studied population according to Sanders’s classification. Twenty-two calcaneal fractures were followed up. However, three CT scans were unretrievable. Most fractures (17/22 = 72%) presented a severe (Sanders III/IV) pattern. The letters (“A,” “B,” “AB,” “AC,” “BC”) inside the bars show the subgroup classification

Twenty-two patients (5 female, 17 male) were included with a mean follow-up of 36 months (± 11). In seven patients (32%), the fracture was work-related. The mean age at injury was 51.2 years (± 10.2). Patient distribution according to the Sanders classification is shown in Fig. 3. Seventy-seven percent of the patients sustained a severe fracture (Type III/IV).

The mean AOFAS score was 72.0 (± 9.8) at follow-up. The mean Böhler angle changed from 12.7° (± 9.5) preoperatively to 28.0° (± 6.3) postoperatively. Table 2 lists the recorded complications. While four (18.2%) wound-healing disorders occurred, no implant-associated surgical revision occurred.

**Table 2:** Complications after intramedullary nailing for calcaneal fractures

Complication	Amount (%)
Wound healing disorders	4 (18.2%)
Requiring revision	2 (9.1%)
Implant associated revisions*	0 (0%)
Local skin irritations	6 (27.3%)
Change of shoes	10 (45.5%)

Wound healing disorders were defined by failed or delayed wound healing with either persistent secretion, reddening, or dehiscence. Four (18.2%) wound healing disorders occurred perioperatively. Two (9.1%) required a single-stage revision. One was an open fracture, and the other was a chronic decubitus. Thus, no implant-associated surgical revisions occurred*. There was no suspicion of implant-associated infections. Local skin irritations were defined as reddening, itching, or blisters occurring in the proximity of the wounds

Table 3 shows the number of patients being pain-free at certain times. Table 4 depicts the patients being able to perform full weight-bearing in relation to time. Tables 5 and 6 show the number of patients being able to return to sports and work at certain times, respectively.

**Table 3:** Fifty percent of patients were pain-free within 1 year

Time to “pain-free”	Amount (%)
< 3 months	6 (27.3%)
< 6 months	8 (36.4%)
< 12 months	11 (50.0%)
> 12 months	17 (77.3%)
Not until follow-up	5 (22.7%)

Seventeen patients (77.3%) were pain-free at follow-up, while 5 remained (22.7%) in pain at follow-up

**Table 4:** Eighty percent of patients were able to perform full weight bearing within 1 year

Return to full weight bearing	Amount (%)
< 6 weeks	1 (9.1%)
< 3 months	4 (19.0%)
< 6 months	16 (40.9%)
< 12 months	18 (81.8%)
> 12 months	21 (95.5%)
Never	0 (0%)
Unknown	1 (4.5%)

Twenty-one patients (95.5%) could perform full weight bearing at follow-up. One patient did not remember when return to full weight bearing was commenced

**Table 5:** More than half of the patients could return to sports within 1 year

Return to sports	Amount (%)
< 3 months	1 (4.5%)
< 6 months	3 (13.6%)
< 12 months	12 (54.5%)
> 12 months	16 (72.7%)
Not until follow-up	6 (27.3%)

Sixteen patients (72.7%) returned to sports at follow-up. 6 patients (27.3%) remained unable to perform sports at follow-up

**Table 6:** Eighty-two percent of patients were able to return to work within 1 year

Return to work	Amount (%)
< 3 months	2 (9.1%)
< 6 months	9 (40.9%)
< 12 months	18 (81.8%)
> 12 months	21 (95.5%)
Not until follow-up	1 (4.5%)

Twenty-one patients (95.5%) could return to work at follow-up. One patient (4.5%) remained unable to work at follow-up

There was a moderate positive correlation (*r* = 0.63, *p* = 0.003) between the time needed to return to full-weight bearing and the time required to return to work. Besides, there was a moderate negative correlation (*r* = − 0.55, *p* = 0.01) between the AOFAS score and the time needed to return to full-weight bearing. There was also a moderate negative correlation (*r* = − 0.62, *p* = 0.003) between the AOFAS score and the time needed to be pain free.

## Discussion

This study examined 22 CFs treated with the C-Nail® implant for a mean follow-up of 36 (± 11) months. The mean AOFAS score was 72.0 (± 9.8). Fifty percent of patients were pain-free within 1 year. Also, within 1 year, 80% of patients could perform full weight-bearing and return to work. Finally, more than half of the patients could return to sports.

In our cohort, several complications occurred. Four patients sustained wound healing disorders, which is important since the minimally-invasive approach was developed to minimize soft tissue damage, wound healing disorders, and infections. Two patients required surgical revision: One patient presented with an open CF that required a single-stage debridement and lavage with implant retainment. The second patient was multi-morbid and needed a revision for a preexisting chronic decubitus at the heel. The two other wound-healing disorders did not require surgical revision. All wound healing disorders were thus classified as “non-implant associated” and did not occur at the site of the incisions. Finally, six patients reported local skin irritations like reddening or itching, which all eventually resolved. No other notable complications occurred. In the literature, equally low rates of wound healing disorders in minimally invasive calcaneal surgery range between 1.9 and 5.2% [23, 26–28, 31]. These low numbers support the use of minimally invasive approaches to minimize wound healing disorders and infections, especially as infection rates can be as high as 24.6% using the conventional far lateral approach [35]. Other reported complications for the Calcanail® and C-Nail® include regional pain syndromes or nerve entrapments and osteomyelitis [36]. None of these was recorded in our population.

The outcome after ankle and heel surgery can be objectified using the AOFAS score [33]. Our patients reported a mean score of 72.0 (± 9.8) at 36 months. Apart from Herlyn et al., who reported a mean score of 71.6 [28], scores in the literature are higher, ranging from 79 [27] to 92.6 [23]. Possible explanations for the lower AOFAS score in our population are the high rate of severe fractures, which usually present worse outcomes. While our cohort contained 77% Sanders III/IV fractures, the cohort studied by Zwipp et al. contained only 31% Sanders III/IV fractures [23]. Besides, some patients might have started to develop subtalar osteoarthritis after 36 months. However, this was not quantified as patients did not receive X-rays or further imaging routinely at follow-up.

Restoration of the Böhler angle is of paramount importance. In this study, the mean Böhler angle changed from 12.7° (± 9.5) preoperatively to 28.0° (± 6.3) postoperatively. This aligns with results published in the literature where preoperative angles range between − 3 and 14° and postoperative angles range between 20 and 33.3° [22, 23, 26–28, 31]. This shows that the procedure using the C-Nail® can restore the joint facet.

Functional outcome, apart from the AOFAS score, is heterogeneously reported in the literature. Fourgeaux et al. reported a mean duration of 6.5 months for the ability to return to work [27], while patients reported by Herlyn et al. only needed 15.8 weeks on average [28]. In our study, return to work levels is reported in time intervals, especially since patients could not remember the exact dates due to the long follow-up. Thus, a direct comparison is not possible. However, at 6 months, only 40.9% of patients returned to work; thus, the mean duration was possibly longer in our population. A possible explanation is the higher severity of fractures in our cohort. Concerning return to sports levels, overall available data is scarce. Herlyn et al. reported that 47% of patients could not return to pre-injury sports levels. In our cohort, only 27.3% could not return to sports activities. Obviously, due to different lengths of follow-up, those results are not comparable. However, Herlyn et al. had a follow-up of 11.3 months, and at this point in time, 54.4% of our patients were already able to return to sports. This contrasts with overall lower AOFAS scores reported by our patients.

Using other minimally invasive approaches, equally high functional levels are achievable. Bischofreiter et al. investigated the functional outcome of 49 patients that received minimally invasive stabilization of CFs using only screws in a technique described by Mattiassich et al. [19]. The studied population consisted of 35% Sanders III/IV fractures. The overall return to sport rate was almost 80% [37].

This study has several limitations: It was a retrospective, singe-center study and patients were operated by only one surgeon. Besides, there was no imaging at follow-up. Furthermore, out of 40 screened CFs, 17 were lost to follow-up, and only 22 were finally included in the analysis. However, this number is even slightly higher than in the study on the C-Nail® implant from Zeman et al. [26]. A major strength of the present study is that the mean follow-up is longer than in most comparable studies using the Calcanail® and C-Nail® [22, 23, 26–31].

## Conclusion

Intramedullary nailing using the C-Nail® implant for severe CFs results in acceptable functional outcomes at mid-terms.

## Data Availability

The data that support the findings of this study are available from the corresponding author, PS, upon reasonable request.
